# How is healthy eating index-2015 related to risk factors for cardiovascular disease in patients with type 2 diabetes

**DOI:** 10.3389/fnut.2023.1201010

**Published:** 2023-05-25

**Authors:** Mobina Zeinalabedini, Ensieh Nasli-Esfahani, Ahmad Esmaillzadeh, Leila Azadbakht

**Affiliations:** ^1^Department of Community Nutrition, School of Nutritional Sciences and Dietetics, Tehran University of Medical Sciences, Tehran, Iran; ^2^Students’ Scientific Research Center (SSRC), Tehran University of Medical Sciences, Tehran, Iran; ^3^Diabetes Research Center, Endocrinology and Metabolism Clinical Sciences Institute, Tehran University of Medical Sciences, Tehran, Iran; ^4^Department of Community Nutrition, School of Nutrition and Food Science, Isfahan University of Medical Science, Isfahan, Iran

**Keywords:** HEI-2015, CVD risk factors, type 2 diabetes, atherogenic index, anthropometric measurement

## Abstract

**Background:**

Cardiovascular disease (CVD) is the primary cause of mortality and disability among diabetes. The aim of this study is to evaluate how healthy eating index-2015 related to risk factors for cardiovascular disease in patients with type 2 diabetes.

**Methods:**

This cross-sectional study was conducted on 490 patients with type 2 diabetes in Tehran, Iran. The healthy eating index-2015 (HEI-2015) used as a diet quality indicator. Dietary intake was assessed by a valid and reliable semi-quantitative food frequency questionnaire (FFQ). Four indicators of CVD risk factor [Castelli risk index-1 and 2 (CRI-II), atherogenic index of plasma (AIP), cholesterol index (CI), and lipid accumulation of plasma (LAP)] were calculated. The anthropometric indices [a body shape index (ABSI), abdominal volume index (AVI), and body roundness index (BRI)] were computed.

**Results:**

After adjusting for potential confounders, it is evident that participants in the highest tertile of HEI had a lower odds ratio of BRI (OR: 0.52; 95% CI: 0.29–0.95; *p*-trend = 0.03) and AIP (OR:0.56; 95% CI: 0.34–0.94; *p*-trend = 0.02). Also, HEI and CRI had a marginally significant negative relation (OR: 0.61; 95% CI: 0.38–1; *p*-trend = 0.05) in crude model, after adjusting the signification disappeared.

**Conclusion:**

In conclusion, our finding shows that more adherence to HEI reduces about 50% of the odds of AIP, BRI among diabetic patients. Further, large-scale cohort studies in Iran need to confirm these findings, including diabetic patients of various racial, ethnic backgrounds, body composition and different components of HEI.

## Introduction

Cardiovascular disease (CVD) is a prominent cause of mortality and morbidity globally (1). As a significant risk factor for CVD, diabetes mellitus (DM) is one of the most rapidly increasing illnesses (2). According to the International Diabetes Federation (IDF), 463 million persons had diabetes in 2019 (3), and it is estimated that 9.2 million Iranians will get diabetes by 2030 (4).

Previous studies have linked diet quality to all-cause mortality and CVD (5, 6). According to estimates, dietary risk factors were responsible for 53% of CVD-related fatalities and 58% of CVD-related disabilities (7). There are few studies on the associations between healthy eating index (HEI) as a diet quality indicator and risk factors for CVD, such as dyslipidemia, diabetes, and obesity.

A body shape index (ABSI) was linked to abdominal fat (8). A high ABSI value suggests a considerable accumulation of adipose tissue around the abdomen area and is a health risk (9). Body Roundness Index (BRI) measures VAT and body fat (10). Obesity, especially visceral obesity, is highly associated with dyslipidemia (11).

DM commonly leads to dyslipidemia raising the risk of arteriosclerotic cardiovascular events (12). Dyslipidemia is characterized by elevated levels of triglycerides (TG), high levels of low-density lipoprotein cholesterol (LDL-C), and reduced levels of high-density lipoprotein cholesterol (HDL-C) (12). Numerous clinical investigations attempt to discover more dependable biochemical indications of atherogenic dyslipidemia (13, 14).

Novel indicators, including lipid ratios, are linked to atherogenesis (15). The atherogenic index of plasma (AIP) effectively predicts myocardial infarction, acute coronary events, atherosclerosis, and CVD morbidity (13). The Cholesterol index (CI) is the most independent predictor and relative risk value for coronary artery disease (16). Lipid accumulation product (LAP) is a simple clinical diagnostic factor for assessing cardiometabolic risk factors used to avoid CVD in the elderly (17).

Castelli risk indexes 1 (CRI-1) and 2 (CRI-2) ratios are independent CAD risk variables with strong predictive value (18, 19). Several studies indicated that greater serum CRI-1 and non-HDL-C were related to an increased risk of stroke (18, 19). Over the last two decades, evidence from observational studies and clinical trials has been collected, emphasizing the value of certain nutrients, foods, and dietary patterns for CVD and DM prevention and treatment (20, 21).

The Healthy Eating Index (HEI) is a measurement of nutritional quality that evaluates how closely one stick to dietary recommendations (20). Based on the findings from a cross-sectional negative correlation between HEI adherence and the development of metabolic syndrome or its components was found (22). The HEI has also been linked to a reduced risk of CVD risk factors (23). According to many studies, those who followed the HEI had a lower risk of CVD and cancer (24–26).

As we know, no research has been conducted on the link between HEI-2015 with novel CVD risk indicators and new anthropometric-related CVD risk factors in type 2 diabetes patients. The purpose of this research is to examine the connection between HEI-2015 with CVD risk variables in diabetic patients.

## Materials and methods

### Study design and population

The main aim of this study is to investigate the relationship between the healthy eating index-2015 (HEI-2015) and food security with CVD risk factors in type 2 diabetes patients in a cross-sectional design. Individuals with type 2 diabetes were recruited from the Tehran University of Medical Sciences affiliated Subspecialty clinic of diabetes and metabolic diseases. Collecting the data was done between May 2021 and September 2022. The participants in this research were 490 randomly chosen people with aged 35–80 referred to a Subspecialty clinic for diabetes and metabolic diseases.

To be eligible, participants needed to meet the following conditions: have type 2 diabetes for at least 2 years, be between the ages of 35 and 80, not be on insulin, not be pregnant or breastfeeding, not be receiving estrogen hormone therapy, and not have an autoimmune disease, acute gastrointestinal disorder, acute renal disease, or liver cancer. Exclusion criteria included a lack of information in the medical records and reporting of dietary energy intake (<800 kcal/day and over 4,200 kcal/day). The research protocol was accepted by the Human Ethical Committee of Tehran University of Medical Science [IR.TUMS.MEDICINE.REC.1400.185].

### Sample size

The sample size for this research was chosen by the prevalence of abdominal obesity in the nation in proportion to the quality of the overall diet as indicated by the index (HEI) (27). Using the formulas P1: the prevalence of abdominal obesity in persons with poor diet quality (0.31), P2: the prevalence of abdominal obesity in individuals with a high diet quality (0.39), $\;C_{1-B}$ = − 0.842, and ca/2 = 1.96, sample size was determined:


$$m^{\prime }=\frac{\;{\left[ca/2\sqrt{(r_{+1}\bar{)p}\bar{Q}}-C_{1-B}\sqrt{r_{1}P_{1}Q_{1}+p_{2}Q_{2}}\right]}^{2}}{r\left(p2-p1\right)2}$$

$$m=\frac{\overset{`}{m}}{4}{\left(1+\sqrt{1+\frac{2\left(r+1\right)}{\overset{`}{m}r\left|P_{2}-P_{1}\right|}}\right)}^{2}$$

$$\bar{P}:\frac{P1+rP2}{r+1}$$

Q1: 1 − P1 = 1 – 0/31 = 0/69 *n*1 = m.

Q2: 1 − P2 = 1 – 0/39 = 0/61 *n*2 = m × *r*.


$$\bar{Q}=1-\bar{P}$$

$$\overset{`}{m}=\frac{\begin{array}{l} \left(1.96\right)\sqrt{\left(\frac{30}{70}+1\right)0.33*0.67}-\\ \left(-0.842\right){\sqrt{\begin{array}{l} \frac{30}{70}*\left(0.31*0.69\right)+\\ \left(0.39*0.61\right) \end{array}}}^{2} \end{array}}{\frac{30}{70}{\left(0.39-0.31\right)}^{2}}=314$$

$$m=\frac{314}{4}{\left(1+\sqrt{1+\frac{2\left(\frac{30}{70}+1\right)}{314\frac{30}{70}\left|0.39-0.31\right|}}\right)}^{2}=345$$

*n*_1_ = m, *n*_2_ = m × *r*

*n*_1_ + *n*_2_ = 345 + 145 = 490

According to this formula, 490 people were needed.

### Data collection

Before the commencement of the study, participants completed written permission forms confirming that they understood and agreed to participate. Those who did not provide informed consent were not included in the study. Patients referred to the clinic must undergo a new laboratory test. As a result, biochemical indicators such as Fasting Blood Sugar (FBS) and LDL-c were obtained from their medical laboratory test. Personal information like age, drug history, taking supplements, and other data were collected in interviews with patients by a dietitian.

### Demographic and socioeconomic status

The socioeconomic status demographic questionnaire, which contained questions about marital status, education, employment, family size, means of support, method of transportation, having a private house, etc., was utilized for this purpose. In order to create the socioeconomic status score, the codes were attached to each questionnaire item. After that, the mean score was reported from 10 to show socioeconomic status score.

### Assessment of dietary intake

An accurate and valid 168-item semi-quantitative food frequency questionnaire (FFQ) was used to estimate typical dietary consumption (28). An experienced nutritionist gathered nutritional data via a face-to-face interview. We calculated the daily intake of each food item and then converted them to grams per day. Then, the Nutritionist IV program was used to calculate macro- and micronutrient intakes (First Databank Division, the Hearst Corporation, San Bruno, CA, United States, modified for Iranian foods).

### Healthy eating index 2015 score

The 2015 HEI contained nine sufficiency components (total fruit, whole fruit, total vegetables, greens and legumes, whole grains, dairy, total protein sources, seafood, plant protein, and fatty acids) and four moderate components (refined grains, sodium, percentage of energy from added sugars, and percentage of energy from saturated fatty acids). Except for the unsaturated to saturated fatty acid ratio, each component is assessed on a density basis out of 1,000 calories. Because several surveys lacked brand specificity and information regarding discretionary salt usage, we changed the sodium component score. Total fruit has 100% fruit juice, while whole fruit includes all forms except juice. Green beans contain legumes, and dairy products include milk. All milk products, such as fluid milk, yogurt, and cheese, as well as fortified soy drinks, are included. Seafood, nuts, seeds, soy products (other than drinks), and legumes are examples of marine food and plant proteins (beans and peas). Based on the distribution of reported salt consumption (mg/d), we classified the individuals into 11 equal groups and awarded corresponding scores ranging from 0 to 10 (higher score for less sodium consumed). Table 1 in the appendix shows the specifics of the HEI-2015 scoring standards. The overall HEI-2015 score ranges from 0 (no adherence) to 100 (complete adherence) (29).

**Table 1:** General characteristics of study participants across tertiles of HEI-2015 (*n* = 490)^1^.

Variables	Tertiles of HEI-2015			*p*^2^
	T_1_ (*n* = 173)	T_2_ (*n* = 160)	T_3_ (*n* = 157)	
Age (y)	61.19 ± 9.56	62.94 ± 10.28	64.00 ± 9.39	0.03
Weight (kg)	74.63 ± 13.30	74.17 ± 11.90	73.02 ± 10.59	0.46
WC (cm)	99.11 ± 10.74	98.45 ± 9.44	97.06 ± 9.34	0.16
HC (cm)	104.77 ± 10.12	105.25 ± 9.97	102.44 ± 9.95	0.02
BMI (kg/m^2^)	28.09 ± 5.41	27.49 ± 4.19	26.94 ± 4.41	0.09
SES (score)	4.55 ± 1.88	4.69 ± 1.98	5.05 ± 1.95	0.07
Female, *n* (%)	99 (57.2%)	101 (63.1%)	90 (57.3%)	0.46
Married, *n* (%)	140 (88.1%)	126 (82.9%)	131 (89.1%)	0.23
Education, *n* (%) (≥diploma)	110 (64.0%)	102 (63.7%)	105 (66.9%)	0.81
Smokers, *n* (%)	17 (9.8%)	12 (7.5%)	14 (8.9%)	0.75
Physically active, *n* (%) (high)	54 (31.2%)	54 (33.8%)	49 (31.2%)	0.98
History of chronic disease, *n* (%)	118 (68.2%)	103 (64.4%)	101 (64.3%)	0.74
History of MACE, *n* (%)	109 (63.0%)	92 (57.5%)	90 (57.3%)	0.48
Supplements user, *n* (%)	63 (36.4%)	76 (47.5%)	85 (54.1%)	0.005
Blood pressure medication, *n* (%)	120 (71.9%)	106 (68.4%)	92 (59.7%)	0.06
Heart disease medication, *n* (%)	57 (34.1%)	47 (30.7%)	49 (31.8%)	0.80
Diabetic diets, *n* (%) (yes)	55 (31.8%)	58 (36.3%)	69 (43.9%)	0.07

WC, waist circumference; HC, hip circumference; BMI, body mass index; SES, socioeconomic status; MACE, major adverse cardiovascular events. ^1^All values are means ± standard deviation (SD), unless indicated. ^2^Obtained from ANOVA for continuous variables and Chi-square test for categorical variables.

### Biochemical indices

In a fasting condition, common laboratory procedures were used to measure fasting blood sugar (FBS), total cholesterol, high-density lipoprotein cholesterol (HDL-c), low-density lipoprotein cholesterol (LDL-c), and triglycerides (TG) (after 12–14 h overnight fasting). In a seated posture, a physician measured the systolic (SBP) and diastolic (DBP) blood pressures using a sphygmomanometer.

### Novel risk factor for cardiovascular disease

By using LDL-C, HDL-C, and TG and following formula the novel risk factor for cardiovascular disease like: CRI-1,CRI-2, LAP, AIP, and CI were obtained:


$$CRI-1=\frac{TC}{HDL-C}$$

$$CRI-2=\frac{LDL-C}{HDL-C}$$

LAP=(WC−65)×(TG)formen

LAP=(WC−58)×(TG)for women

CI=IFTF>400(LDL−HDL+1.5)×TG

CI=IFTG<400(LDL−HDL)×TG

$$AIP=\log\;\frac{TG}{HDL-C}$$

### Anthropometric indices

Patients were weighed in light clothes and barefoot on a digital scale (SECA, Hamburg, Germany) to the nearest 0.1 kg. Standing height was measured using a stadiometer set on the wall, with accuracy to within 0.5 cm. Using a measuring tape, determine the waist circumference (WC) of the patient at the narrowest point, immediately above the belly button. A dietician measured the hip circumferences of patients by placing a tape measure around the widest part of the hips.

The following formulae were used to determine body mass index (BMI):


$$BMI=\frac{Weight}{{\left(height\right)}^{2}}$$

Body mass index is calculated as kg/m^2^. ABSI and BRI and abdominal volume index (AVI) were computed using previously described methods using WC (m), BMI (kg/m^2^), and height (m) as below:


ABSI=WCBMI23×height12

$$BRI=364.2-365.5\times \sqrt{1-(\frac{{\left(\frac{WC}{2\pi }\right)}^{2}}{{\left(0.5height\right)}^{2}}})$$

$$AVI=\frac{2\;{\left(WC\right)}^{2}+0.7\;{\left(WC-hip\right)}^{2}}{1000}$$

### Physical activity

We utilized the International Physical Activity Questionnaire (IPAQ) short form to measure physical activity levels (30). The IPAQ consisted of seven questions. Overall, the questions assessed the number of days and minutes spent participating in light and heavy activities, as well as the average time spent walking and sitting during the previous 7 days. Physical activity levels of 600 METs-min/week were classed as low, 600–3,000 METs-min/week as moderate, and > 3,000 METs-min/week as high.

### Other variables

We considered Five-point MACE for this study. Myocardial infarction, stroke, hospitalization for heart failure, and revascularization treatments, including angioplasty and bypass surgery, are all included in the definition of Major Adverse Cardiac Events (MACE). Since we do not have CV death for our study population, we put it aside.

Patients were asked about their history of chronic diseases. Chronic disease history includes Thyroid, blood pressure, cardiovascular disease, chronic kidney disease, and chronic obstructive pulmonary disease.

### Statistical analysis

First of all, subjects were classified based on tertiles of HEI-2015 scores (T1: 43–59; T2: 60–66; T3: 67–88). Baseline quantitative and qualitative variables were compared across HEI tertiles by Analysis of Variance (ANOVA) and Chi-square tests, respectively. Continuous variables were reported as mean (SD), and categorical variables were shown as numbers and percentages. Dietary intakes of HEI components and other nutrients were evaluated across HEI tertiles using Analysis of Covariance (ANCOVA). In this regard, total energy and macronutrient intakes were adjusted for age and gender, but other nutrients were additionally controlled for energy (Kcal). The associations of HEI with CVD risk factors and anthropometric indices were explored through binary logistic regression. Based on previous investigations (31, 32), we considered ABSI, BRI, AVI, and AIP cut-off points to be 0.08, 5.20, 17.30, and 0.11, respectively. Values above the mentioned cut points were considered as the dependent variable in this study. For other CVD risk factors (CRI-1, CRI-2, Cholindex, and LAP), participants were categorized into tertiles of each variable. As there is no specific cut-off point for these variables, the last tertile of each risk factor was considered the dependent variable in the logistic regression. Odds ratios (ORs) and 95% confidence intervals (95% CIs) were reported for the association of HEI with CVD risk factors and anthropometric indices in crude and adjusted models. In case of anthropometric indices, two multivariable-adjusted models were applied. For the first model, we controlled the confounding effects of age, gender. and energy intake. The second model was further adjusted for smoking, socioeconomic status, chronic disease history, MACE history, diabetic diets, education level, marital status, physical activity, and supplement use. Considering CVD risk factors, we also included blood pressure and heart disease medications in the second model.

Furthermore, an additional model (model 3) was considered for CVD risk factors in order to control the confounding effect of BMI as well. The first tertile of HEI was considered the reference for all analyses. The trend of ORs across tertiles of HEI was obtained by considering HEI tertiles as an ordinal variable in the logistic regression models. All statistical analyses were performed by SPSS software (version 26; SPSS Inc., Chicago, IL). *p* < 0.05 was considered a significant level.

## Results

### Patients’ socio-demographic characteristics

The study included 490 adults in total. Table 1 displays patient socio-demographic characteristics based on HEI-2015 tertiles. The mean age of the patients across the tertiles was 61.19, 62.94, and 64 years (*p* = 0.03). The tertiles 1 group has the highest BMI, with a mean of 28.09 kg/m^2^. Most variables had no significant differences between tertiles except for hip circumference (*p* = 0.02) and supplement user (*p* = 0.005). Of 490 individuals, 66.8% of patients used blood pressure medication, and just 37.1% followed a diabetic diet.

### Biochemical and anthropometric indices of participants

Table 2 describes patients’ biochemical, novel CVD risk factors, and anthropometric indices. SBP and DBP averaged 125.44 ± 15.17 mmHg and 77.89 ± 7.88 mmHg, respectively. The first tertile had the highest FBS and HbA1C, with mean values of 157.53 mg/dL and 8.17%, respectively. There are significant differences in BRI (*p* = 0.04) but only marginally in LAP (*p* = 0.07).

**Table 2:** Biochemical and anthropometric indices of study participants across tertiles of HEI-2015 (*n* = 490)^1^.

Variables	Tertiles of HEI-2015			*p*^2^
	T_1_ (*n* = 173)	T_2_ (*n* = 160)	T_3_ (*n* = 157)	
SBP (mmHg)	124.86 ± 14.17	127.02 ± 15.17	124.45 ± 16.76	0.25
DBP (mmHg)	78.16 ± 6.63	77.56 ± 7.50	77.93 ± 9.42	0.78
FBS (mg/dl)	157.53 ± 56.02	155.93 ± 64.89	154.59 ± 72.34	0.91
HbA_1_C (%)	8.17 ± 1.75	8.03 ± 1.69	7.85 ± 1.82	0.25
TC (mg/dl)	153.01 ± 37.18	147.19 ± 36.66	148.20 ± 37.53	0.30
TG (mg/dl)	145.188 ± 62.72	145.09 ± 73.18	133.36 ± 52.88	0.16
HDL-C (mg/dl)	44.68 ± 10.78	43.31 ± 10.72	43.75 ± 10.35	0.48
LDL-C (mg/dl)	78.70 ± 25.84	75.25 ± 26.76	77.14 ± 27.76	0.50
ABSI (m1^1/6^ kg^−2/3^)	0.08 ± 0.008	0.08 ± 005	0.08 ± 0.006	0.98
BRI	5.76 ± 1.48	5.57 ± 0.97	5.43 ± 1.06	0.04
AVI	19.92 ± 4.33	19.61 ± 3.70	19.05 ± 3.61	0.12
CRI-I	3.48 ± 0.90	3.42 ± 0.93	3.42 ± 0.91	0.43
CRI-II	1.79 ± 0.66	1.73 ± 0.72	1.75 ± 0.69	0.62
CI	0.87 ± 0.64	0.83 ± 0.66	0.86 ± 0.68	0.81
AIP	0.12 ± 0.21	0.13 ± 0.23	0.10 ± 0.20	0.58
LAP	63.12 ± 35.25	61.75 ± 35.50	55.15 ± 27.78	0.07

SBP, systolic blood pressure; DBP, diastolic blood pressure; FBS, fasting blood sugar; HbA_1_C, hemoglobin A_1_C; TC, total cholesterol; TG, triglycerides; HDL-C, high density lipoprotein cholesterol; LDL-C, low density lipoprotein cholesterol; ABSI, a body shape index; BRI, body roundness index; AVI, abdominal volume index; CRI-1, castelli index-I; CRI-II, castelli index-II; CI, cholestrol index; AIP, atherogenic index of plasma; LAP, lipid accumulation product. ^1^All values are means ± standard deviation (SD), unless indicated. ^2^Obtained from ANOVA for continuous variables.

### Nutrients and food groups

Significant differences (*p* < 0.001) were observed in the weighted proportions of participants who received the highest component score on the HEI-2015 component. There was also identification of the participant who received the least sodium (*p* = 0.05; Table 3).

**Table 3:** Multivariable-adjusted intakes of selected nutrients and food groups of study participants across tertiles of HEI-2015 (*n* = 490)^1^.

Variables	Tertiles of HEI-2015			*p*^2^
	T_1_ (*n* = 173)	T_2_ (*n* = 160)	T_3_ (*n* = 157)	
Energy (Kcal/d)	1678.90 ± 34.26	1670.12 ± 35.41	1583.34 ± 35.82	0.111
**HEI-2015 components**				
Whole grains (Oz per 1,000 Kcal)	0.36± 0.04	0.88 ± 0.04	1.41 ± 0.04	<0.001
Refined grains (Oz per 1,000 Kcal)	6.36 ± 0.14	5.37 ± 0.14	4.64 ± 0.15	<0.001
Seafood and plant proteins (cups per 1,000 Kcal)	2.06 ± 0.052	2.24 ± 0.054	2.36 ± 0.054	<0.001
Sodium (grams per 1,000 kcal)	4.55 ±0.12	4.36 ± 0.12	4.79 ± 0.12	0.05
Dairy (cups per 1,000 kcal)	0.79 ± 0.03	0.93 ± 0.03	0.95 ± 0.03	<0.001
Greens and beans (cups per 1,000 kcal)	0.35 ±0.01	0.42 ± 0.01	0.44 ± 0.01	<0.001
Total vegetable (cups per 1,000 kcal)	1.39 ± 0.04	1.53 ± 0.04	1.74 ±0.04	<0.001
Whole fruit (cups per 1,000 kcal)	1.38 ± 0.03	1.55 ± 0.03	1.66 ± 0.03	<0.001
Total fruit (cups per 1,000 kcal)	1.41 ± 0.03	1.57 ± 0.03	1.67 ± 0.03	<0.001
Added sugar (% of energy)	5.87 ± 0.24	4.84 ± 0.25	3.50 ± 0.25	<0.001
MUFA/PUFA. ratio	1.76 ± 0.03	1.96± 0.03	2.27 ± 0.03	<0.001
Saturated fats (% of energy)	10.73 ± 0.16	9.82 ± 0.16	8.90 ± 0.16	<0.001
**Macronutrients**				
Carbohydrates (% of energy)	56.13 ± 0.40	56.17 ± 0.42	56.03 ± 0.42	0.97
Proteins (% of energy)	12.32 ± 0.12	13.02 ± 0.12	13.47 ± 0.12	<0.001
Fats (% of energy)	34.00 ± 0.40	33.47 ± 0.42	33.21 ± 0.42	0.39
**Other nutrients**				
Cholesterol (mg/d)	187.82 ± 6.09	198.37 ± 6.29	178.53 ± 6.38	0.087
Calcium (mg/d)	746.58 ± 16.02	846.26 ± 16.55	861.88 ± 16.78	<0.001
Selenium (mg/d)	0.05 ± 0.00	0.05 ± 0.00	0.05 ± 0.00	0.47
Iron (mg/d)	12.80 ± 0.19	13.03 ± 0.20	12.77 ± 0.20	0.61
Magnesium (mg/d)	198.69 ± 2.37	219.13 ± 2.44	226.02 ± 2.48	<0.001
Fiber (g/d)	13.38 ± 0.23	14.68 ± 0.23	15.95 ± 0.24	<0.001
Vitamin B1 (mg/d)	1.27 ± 0.01	1.26 ± 0.01	1.31 ± 0.01	0.08
Vitamin B2 (mg/d)	1.33 ± 0.02	1.48 ± 0.02	1.48 ± 0.02	<0.001
Vitamin B3 (mg/d)	13.63 ± 0.18	13.50 ± 0.18	14.06 ± 0.19	0.09
Vitamin B6 (mg/d)	1.15 ± 0.02	1.18 ± 0.02	1.18 ± 0.02	0.56
Vitamin B9 (mcg/d)	234.87 ± 4.25	255.78 ± 4.39	262.51 ± 4.45	<0.001
Vitamin B12 (mcg/d)	2.58 ± 0.07	2.86 ± 0.07	2.91 ± 0.07	0.003

^1^All values are means ± standard error (SE); total energy and macronutrients intake are adjusted for age and gender; all other values are adjusted for age, gender, and energy intake. ^2^Obtained from ANCOVA.

Participants in the highest tertile of the HEI-2015 consumed more protein (*p* = 0.001), calcium, magnesium, fiber, vitamin B2, and vitamin B9 (*p* < 0.001). There are no significant differences in the intake of certain nutrients, such as selenium (*p* = 0.47), vitamin B6 (*p* = 0.55), and iron (*p* = 0.61; Table 3).

**Table 4:** Multivariable- adjusted odds ratio novel CVD risk factors across tertiles of HEI-2015 (*n* = 490)^1^.

Variables	Tertiles of HEI-2015			*p*-trend
	T_1_ (*n* = 173)	T_2_ (*n* = 160)	T_3_ (*n* = 157)	
**AIP**				
Crude	1.00	0.51 (0.32–0.82)	0.54 (0.34–0.87)	0.01
Model 1	1.00	0.53 (0.33–0.86)	0.56 (0.34–0.90)	0.01
Model 2	1.00	0.54 (0.33–0.88)	0.56 (0.33–0.93)	0.02
Model 3	1.00	0.55 (0.33–0.89)	0.56 (0.34–0.94)	0.02
**Choline**				
Crude	1.00	0.84 (0.52–1.37)	0.94 (0.58–1.52)	0.81
Model 1	1.00	0.88 (0.53–1.45)	1.10 (0.67–1.80)	0.71
Model 2	1.00	0.90 (0.54–1.50)	1.04 (0.62–1.75)	0.88
Model 3	1.00	0.95 (0.57–1.59)	1.09 (0.64–1.84)	0.76
**CRI-1**				
Crude	1.00	0.83 (0.51–1.34)	0.61 (0.38–1.00)	0.05
Model 1	1.00	0.89 (0.55–1.46)	0.69 (0.41–1.14)	0.15
Model 2	1.00	0.93 (0.56–1.54)	0.67 (0.39–1.15)	0.16
Model 3	1.00	0.97 (0.59–1.162)	0.96 (0.40–1.19)	0.20
**CRI-2**				
Crude	1.00	0.87 (0.54–1.41)	0.76 (0.47–1.23)	0.27
Model 1	1.00	0.93 (0.57–1.51)	0.86 (0.53–1.42)	0.58
Model 2	1.00	0.96 (0.58–1.58)	0.77 (0.46–1.31)	0.36
Model 3	1.00	0.97 (0.59–1.61)	0.78 (0.46–1.34)	0.39
**LAP**				
Crude	1.00	1.04 (0.64–1.69)	0.74 (0.45–1.22)	0.26
Model 1	1.00	1.02 (0.62–1.67)	0.75 (0.45–1.24)	0.28
Model 2	1.00	1.08 (0.65–1.77)	0.84 (0.49–1.43)	0.57
Model 3	1.00	1.20 (0.71–2.03)	0.90 (0.51–1.59)	0.77

AIP, atherogenic index of plasma; CRI-1, Castelli risk index-1; CRI-2, Castelli risk index 2; LAP, lipid accumulation product. Model 1: adjusted for age, gender, energy intake. Model 2: further adjustment for smoking, socioeconomic status, chronic disease history, history of MACE, having diabetic diets, education, marital status, physical activity, blood pressure medications, heart disease medications, and supplement use. Model 3: more adjustments for BMI. ^1^All values are odds ratios and 95% confidence intervals.

### Cardiovascular risk factor and association with healthy eating index

In the crude model and after adjusting for potential confounders such as age, energy, smoking, welfare, CDH, diabetic diet, BP medication, and heart medication, patients who had more adherence to HEI had lower AIP (OR: 0.56; 95% CI: 0.34–0.94; *p*-trend =0.02) (Table 4).

**Table 5:** Multivariable- adjusted odds ratio novel anthropometrics across tertiles of HEI-2015 (*n* = 490)^1^.

Variables	Tertiles of HEI-2015			*p*-trend
	T_1_ (*n* = 173)	T_2_ (*n* = 160)	T_3_ (*n* = 157)	
**ABSI**				
Crude	1.00	1.54 (0.8–2.64)	1.20 (0.71–2.01)	0.47
Model 1	1.00	1.50 (0.86–2.61)	1.03 (0.60–1.77)	0.84
Model 2	1.00	1.62 (0.92–2.85)	1.17 (0.66–2.07)	0.52
**AVI**				
Crude	1.00	1.15 (0.69–1.91)	0.82 (0.50–1.34)	0.44
Model 1	1.00	1.13 (0.68–1.89)	0.81 (0.49–1.34)	0.43
Model 2	1.00	1.91 (0.70–2.01)	0.98 (0.57–1.68)	0.97
**BRI**				
Crude	1.00	0.88 (0.55–1.42)	0.59 (0.37–0.94)	0.02
Model 1	1.00	0.69 (0.39–1.21)	0.42 (0.24–0.74)	0.003
Model 2	1.00	0.73 (0.41–1.30)	0.52 (0.29–0.95)	0.03

ABSI, a body shape index; AVI, abdominal volume index; BRI, body roundness index. Model 1: adjusted for age, gender, energy intake. Model 2: further adjustment for smoking, socioeconomic status, chronic disease history, history of MACE, having diabetic diets, education, marital status, physical activity, and supplement use. ^1^All values are odds ratios and 95% confidence intervals.

For CRI-I, participants with more adherence to HEI had a lower odds ratio of CRI-I (OR: 0.61; 95% CI: 0.38–1.00; *p*-trend = 0.05), although this signification disappeared after the adjustment.

### Anthropometric indices and association with healthy eating index

Participants who had higher adherence to HEI-2015 were 59% fewer odds of BRI (95% CI: 0.37–0.94; *p*-trend = 0.02), and this association was still significant after adjusting for all the confounders (OR: 0.52; 95% CI: 0.29–0.95; *p*-trend = 0.03). There was no signification in other anthropometric indices like; ABSI and AVI (Table 5).

## Discussion

In the present cross-sectional study of Iranian adult patients with type 2 diabetes, we discovered a significant positive relationship between maximal adherence to HEI-2015 and lower CRI-1 just in the crude model, as well as a significant negative relationship between greater adherence to HEI-2015 and lower odds of AIP and BRI.

Microvascular dysfunction was shown to be connected with type 2 diabetes, and the likelihood of this condition was further raised by being overweight (33). One concept is central obesity, and visceral fat has a role in developing CVD (34). The consequences of excessive adiposity on the myocardial and vascular have a direct impact on cardiac function (35).

The body roundness index (BRI) was created to evaluate the form of the human body figure as an ellipse in order to forecast both body fat and the proportion of visceral adipose tissue using WC in relation to height, which might be reduced by greater adhering to HEI (33, 36, 37). Improved dietary quality has been linked to a reduced prevalence of CVD risk factors (38).

The HEI Index measures the quality of food based on the dietary habits of the US population, and the highest score of HEI is for more consumption of healthy nutrients and <8% saturated fat, sodium, and added sugar (29). According to a previous study, abdominal fat may accumulate in response to higher dietary SFA intakes (39).

Therefore, greater adherence to HEI-2015 could enormously improve abdominal obesity. Although we could not find a significant statically relation to other anthropometric indices but clinically higher HEI might reduce abdominal obesity, which aligns with other studies’ results (40–42). In contrast, some studies were inconsistent with our results, and several failed to observe any association (43, 44).

Researchers have identified plasma lipid profiles as a significant risk factor and predictor of cardiovascular disease (CVD) in recent years (13). Atherosclerosis is brought on by dyslipidemia, characterized by a rise in low-density lipoprotein cholesterol (LDL-C), total cholesterol, triglycerides (TG), and a decrease in HDL-C (45). LDL-C was formerly thought to be a primary therapy goal. Yet, once LDL-C dropped to the required levels, around half of the remaining cardiovascular risks persisted, leading researchers to identify new CVD predictors (46).

The atherogenic index of plasma (AIP) (47) has been proposed as a marker of plasma atherogenicity in addition to individual serum cholesterol levels, including LDL-C, due to its positive association with the lipoprotein particle size, cholesterol esterification rates, and remnant lipoproteinemia (48, 49). In addition to being a strong predictor of atherosclerosis and coronary heart disease, AIP also faithfully portrays the connection between protective and atherogenic lipoproteins, especially in type 2 diabetes (50).

Our findings showed that HEI-2015 was negatively associated with AIP and that a higher HEI-2015 score corresponds to a larger diet of nuts and beans and a lower intake of SFA. Although Altamimi et al. (51) reported that nuts, particularly walnuts, hazelnuts, and pistachios, lead to an effective increase in HDL levels, Nora et al. (52) found no significant difference in TC, HDL, and LDL in the group that consumed 69 g nuts/day compared to the control group in a 16-week study of obese and overweight people.

The content of isoflavones and lecithin in soy and nuts can cause a decrease in LDL and an increase in HDL. Isoflavones may increase HDL-cholesterol production in the liver and ameliorate dyslipidemia by suppressing adipogenesis (53) and activating lipoprotein lipase (54).

According to our findings, adherence to a healthy eating pattern decreased about 40% of the risk of CRI-1. This signification vanished after adjusting by potential confounders. This indicator is based on three significant lipid profile parameters: TC, LDL-C, and HDL-C, and it’s determined as the ratio of TC/HDL-C. Since CRI-1 reflect the modification of a crucial component of vascular risk—the decline in levels of the protective cholesterol fraction, HDL-C, at the expense of the rise in TC and specifically the risk fraction, LDL-C—they have a predictive value for CVD that is greater than that of the isolated data of the lipid profile (55).

This might be because a higher intake of vegetables, fruits, whole grains, low-fat dairy products, and total protein, which is in a higher score HEI, could strongly improve lipid profile (23, 56). Also, Studies showed that a higher score of HEI is strongly related to higher HDL, which could be decreased the risk of CRI-1 (22, 56, 57). However, Rashidipour-Fard et al. found a statistically significant positive correlation between HDL-C and HEI scores in their cross-sectional study. However, after accounting for age, sex, calorie consumption, and BMI, it changed to a non-significant level (23).

The current studies have many strengths. To the best of our knowledge, this is the first observational study investigating the relationship between HEI and novel anthropometrics-related CVD risk factors and CVD risk factors among type 2 diabetes. The sample size of this research is sufficient to support our findings. Also, we used a valid and accurate FFQ to gather nutritional data, adjusting for many possible confounders, and in-depth interviews with participants conducted by professional nutritionists.

There is a considerable limitation that could affect our result. Firstly, we have not seen the odds of the ratio of CVD risk factors in different components of HEI groups. Also, the healthy eating index is a proxy for overall quality and does not account for quantity. It is impossible to characterize a causality relationship given the study’s cross-sectional design.

In conclusion, our finding shows that adherence to HEI-2015 reduces about 50% of the risk of AIP and BRI among diabetic patients. Further, these findings need to be confirmed by large-scale cohort studies in Iran that include patients of various racial and ethnic backgrounds. In addition, consider the patient’s body composition and, in addition, different components of HEI to establish the relationship between HEI and CVD risk factors.

## Data availability statement

The raw data supporting the conclusions of this article will be made available by the authors, without undue reservation.

## Ethics statement

The studies involving human participants were reviewed and approved by the Human Ethical Committee of Tehran University of Medical Science [IR.TUMS.MEDICINE.REC.1400.185]. The patients/participants provided their written informed consent to participate in this study.

## Author contributions

MZ, LA, AE, and EN-E designed the study. EN-E supported the analysis of the CVD risk factors. MZ carried out the study and analyzed the data. MZ and LA interpreted the findings, drafted the manuscript, and revised the final manuscript. AE commented on the presentation of data and his comments improved the quality of the paper significantly. He also commented on the different parts of the study and reviewed the paper scientifically and edited the paper for language errors. All authors contributed to the article and approved the submitted version.

## Funding

This study was supported by the Tehran University of Medical Sciences (grant number: 9911468002).

## Conflict of interest

The authors declare that the research was conducted in the absence of any commercial or financial relationships that could be construed as a potential conflict of interest.

## Publisher’s note

All claims expressed in this article are solely those of the authors and do not necessarily represent those of their affiliated organizations, or those of the publisher, the editors and the reviewers. Any product that may be evaluated in this article, or claim that may be made by its manufacturer, is not guaranteed or endorsed by the publisher.
