# Supplementary material for: How is healthy eating index-2015 related to risk factors for cardiovascular disease in patients with type 2 diabetes
Source: Front Nutr. 2023 May 25;10:1201010. doi: 10.3389/fnut.2023.1201010 (PMC10248502; doi:10.3389/fnut.2023.1201010)
Supplement: Supplementary file 1 [file Table_1.DOCX]

| Component | Max  point | Maximum point standard | Minimum point standard |
| --- | --- | --- | --- |
| Sufficient | | | |
| Total fruit | 5 | ≥0.8 cups | No intake |
| Whole fruit | 5 | ≥0.4 cups | No intake |
| Total vegetable | 5 | ≥1.1 cups | No intake |
| Greens and beans | 5 | ≥0.2 cups | No intake |
| Whole grains | 10 | ≥1.5 cups | No intake |
| Dairy | 10 | ≥1.3 cups | No intake |
| Total Protein foods | 5 | ≥2.5 cups | No intake |
| Seafood and plant proteins | 5 | ≥0.8 cups | No intake |
| Fatty acids | 10 | (PUFAs+MUFAs)/SFAs≥2.5 | (PUFAs+MUFAs)/SFAs≤1.2 |
| Moderate | | | |
| Refined Grains | 10 | ≤1.8 oz | ≥4.3 oz |
| Sodium | 10 | Maximum decile | Minimum decile |
| Added Sugars | 10 | ≤6.5% of energy | ≥26% of energy |
| Saturated Fats | 10 | ≤8% of energy | ≥16% of energy |

Table 1. Components and Scoring Guidelines for the HEI-2015
